# Does Motor Cortex Engagement During Movement Preparation Differentially Inhibit Nociceptive Processing in Patients with Chronic Whiplash Associated Disorders, Chronic Fatigue Syndrome and Healthy Controls? An Experimental Study

**DOI:** 10.3390/jcm9051520

**Published:** 2020-05-18

**Authors:** Lisa Goudman, André Mouraux, Liesbeth Daenen, Jo Nijs, Patrick Cras, Nathalie Roussel, Maarten Moens, Dorine Lenoir, Iris Coppieters, Eva Huysmans, Margot De Kooning

**Affiliations:** 1 Department of Neurosurgery, Universitair Ziekenhuis Brussel, 1090 Brussels, Belgium; lisa.goudman@vub.be (L.G.); Maarten.Moens@uzbrussel.be (M.M.); 2 Pain in Motion International Research Group, www.paininmotion.be, Brussels, Belgium; Liesbeth.Daenen@idewe.be (L.D.); jo.nijs@vub.be (J.N.); dorine.lenoir@ugent.be (D.L.); iris.coppieters@vub.be (I.C.); eva.huysmans@vub.be (E.H.); 3 Department of Physiotherapy, Human Physiology and Anatomy, Faculty of Physical Education & Physiotherapy, Vrije Universiteit Brussel, 1090 Brussels, Belgium; 4 Institute of Neuroscience, Université catholique de Louvain, 1200 Brussels, Belgium; andre.mouraux@uclouvain.be; 5 Knowledge, Information and Research Center (KIR), Group Idewe, 3001 Louvain, Belgium; 6 Department of Physical Medicine and Physiotherapy, Universitair Ziekenhuis Brussel, 1090 Brussels, Belgium; 7 Laboratory of Neurology, Translational Neurosciences, University of Antwerp, 2610 Wilrijk, Belgium; patrick.cras@uantwerpen.be; 8 Institute Born-Bunge, University of Antwerp, 2610 Wilrijk, Belgium; 9 Department of Neurology, Antwerp University Hospital,2650 Edegem, Belgium; 10 Department of Rehabilitation Sciences and Physiotherapy (MOVANT), Faculty of Medicine and Health Sciences, University of Antwerp, Campus Drie Eiken, 2610 Wilrijk, Belgium; nathalie.roussel@uantwerpen.be; 11 Center for Neurosciences (C4N), Vrije Universiteit Brussel (VUB), 1090 Brussels, Belgium; 12 Department of Radiology, Universitair Ziekenhuis Brussel, 1090 Brussels, Belgium; 13 Department of Rehabilitation Sciences and Physiotherapy, Ghent University, 9000 Ghent, Belgium; 14 Department of Public Health (GEWE), Faculty of Medicine and Pharmacy, Vrije Universiteit Brussel, 1090 Brussels, Belgium

**Keywords:** Laser-evoked potentials, exercise-induced hypoalgesia, chronic pain, EEG

## Abstract

Background: Patients with chronic fatigue syndrome (CFS) and chronic whiplash associated disorders (cWAD) present a reduced ability to activate central descending nociceptive inhibition after exercise, compared to measurements before exercise. It was hypothesised that a dysfunctional motor-induced inhibition of nociception partly explains this dysfunctional exercise-induced hypoalgesia. This study investigates if engagement of the motor system during movement preparation inhibits nociception-evoked brain responses in these patients as compared to healthy controls (HC). Methods: The experiment used laser-evoked potentials (LEPs) during three conditions (no task, mental task, movement preparation) while recording brain activity with a 32-channel electroencephalogram in 21 patients with cWAD, 20 patients with CFS and 18 HC. Two-factor mixed design Analysis of variance were used to evaluate differences in LEP amplitudes and latencies. Results: No differences in N1, N2, N2P2, and P2 LEP amplitudes were found between the HC, CFS, and cWAD groups. After nociceptive stimulation, N1, N2 (only at hand location), N2P2, and P2 LEP amplitudes significantly decreased during movement preparation compared to no task (within group differences). Conclusion: Movement preparation induces a similar attenuation of LEPs in patients with CFS, patients with cWAD and HC. These findings do not support reduced motor-induced nociceptive inhibition in these patients.

## 1. Introduction

The experience of pain can limit the ability to perform motor tasks, which is caused by nociception–motor interactions [1]. It has been demonstrated that proprioception, force steadiness, muscle activity and coordination are altered by the experimental induction of pain [2,3]. Further, the level of pain sensitivity can be decreased by motor cortex stimulation through epidural electrodes, repetitive transcranial magnetic stimulation, or performance of physical exercise [4,5,6,7,8]. Engagement of the motor cortex however does not only occur when carrying out movements, but also during the movement-preparation phase [9,10,11]. Previous research has indicated that engagement of the primary motor cortex during movement preparation reduces pain and nociception-evoked potentials, suggesting that engagement of the motor cortex exerts an inhibitory effect on nociception [12].

Two syndromes with a generalised hyper-responsiveness of the central nervous system to a variety of stimuli are chronic fatigue syndrome (CFS) and chronic whiplash associated disorders (cWAD). Patients with cWAD suffer from a variety of clinical manifestations such as pain, fatigue, concentration difficulties, and headaches [13,14]. CFS is characterised by a medically unexplained disabling fatigue that persists for more than 6 months (primary symptom) [15]. Besides fatigue, also multi-joint pain, impaired concentration, and post-exertional malaise are frequently occurring [16,17]. The influence of pain on motor function in chronic pain patients can also be seen in the dysfunctional responses to exercise. These patients appear to exhibit reduced central descending nociceptive inhibition while performing exercises (assumed due to differences in measurements before and after exercise), which is normally seen in healthy controls [18,19]. Up to now, the exact mechanism of this impaired exercise-induced hypoalgesia is unknown. However, it is suggested that an impaired inhibition of nociception induced by the motor cortex can possibly partly explain this dysfunctional response [20].

De Pauw et al. (2019) tested the hypothesis that changes in brain morphology are an underlying process of motor impairment in patients with WAD. They revealed a decrease in gray matter volume of the precentral gyrus, which is part of the motor cortex, compared to healthy controls. Additionally, an association was found between the volume of the precentral gyrus and both neuromuscular control and strength [21]. Another study evaluated motor cortical excitability in patients with CFS during repetitive finger movements. It was found that patients with CFS do not show normal fluctuations of motor cortical excitability during and after the exercise [22]. Additionally, patients with CFS might have a deficit in motor preparatory areas of the brain, a hypothesis that was developed based on the slowness of simple reaction times in these patients [23].

Evaluating whether nociception–motor interactions are disrupted in patients with CFS and cWAD, has not yet been performed. To address this existing gap in knowledge, the goal of this study was to investigate if motor cortex engagement during movement preparation inhibits the cortical responses to nociceptive stimuli in patients with CFS and patients with cWAD compared to healthy controls. Nociceptive laser stimuli were used as nociceptive stimuli. Laser-evoked potentials (LEPs) can be used to conduct a functional evaluation of the nociceptive afferent pathways [24]. Up to now, findings regarding LEPs in patients with enhanced pain sensitivity are still inconclusive. However, in some chronic pain patients, enhanced LEPs are suggested to be related to central sensitisation, at least in part [25,26]. In patients with fibromyalgia, studies already reported increased amplitudes of the N2 and P2 waves of LEPs [27]. In this study, it is hypothesised that movement preparation would only result in an attenuation of LEP amplitudes (i.e., inhibitory effect on nociceptive system) in healthy controls and not in-patient populations (impaired exercise-induced hypoalgesia). Therefore, the main outcome measure was the difference in LEP N2P2 amplitude between rest and movement preparation in both patient populations. Similarly, it was hypothesised that pain intensity ratings for nociceptive stimuli during movement preparation would be reduced in healthy controls and not in-patient population. In addition to comparing the elicited responses in the time domain, a time-frequency analysis was conducted to characterize transient stimulus-evoked modulations of oscillatory electroencephalogram activity. Therefore, an experiment was set-up using laser-evoked potentials (LEPs) in 62 study participants (patients with cWAD, patients with CFS and healthy controls (HC)).

## 2. Materials and Methods

### 2.1. Participants

Twenty-one patients with cWAD, 21 patients with CFS and 20 HC participated in this study. Before study participation, all patients provided written informed consent. The study was conducted according to the revised Declaration of Helsinki (1998). Approval of the study protocol was obtained from the Ethics Committee of the University of Antwerp (approval number B300201214521).

Patients were recruited through advertisement on the website of Pain in Motion (international research group; http://www.paininmotion.be), from a medical database obtained from previous studies, from the medical database of the local Red Cross medical care unit (for patients with cWAD) and via private physician practices (for patients with CFS). Patients with cWAD were only eligible for inclusion if they experienced chronic symptoms resulting from a whiplash trauma (e.g., motor vehicle accident or fall) and fulfilling diagnostic criteria of WAD grade I to III as defined by the Quebec Task Force classification [28]. Chronicity was defined as complaints persisting for at least 3 months. Patients who were classified as WAD grade IV [28], were excluded. Patients with CFS were only eligible if they were diagnosed by a physician, according to the 1994 Center for Disease Control and Prevention criteria [15]. This implies that any other medical condition (cardiovascular, neurological, psychiatric or haematological) possibly explaining the debilitating fatigue and pain was excluded prior to establishing the diagnosis of CFS.

Healthy controls were recruited among the university college staff, family members and acquaintances of the researchers. Participants were not eligible if they previously experienced a whiplash trauma, suffered from persistent pain or neck-shoulder-arm symptoms, or had sought medical help for neck-shoulder-arm symptoms in the past 6 months. Additionally, healthy participants were excluded if they were suffering from an acute or chronic disease, or when they were experiencing pain on the day of the assessment.

Participants were excluded if they were pregnant, or if they suffered from any cardiovascular or neurological disease. All participants were asked to discontinue non-opioid analgesic and anti-inflammatory drugs 48 h before testing. Additionally, participants were asked to avoid physical exertion and to refrain from consuming nicotine, alcohol and caffeine 24 h before the assessment. To limit confounding of the study findings, we aimed to recruit 3 groups with a comparable age distribution.

Demographics of the 3 groups were compared with Kruskal-Wallis tests and chi-squared tests, depending on the normality and variance of the data.

### 2.2. Experimental Design

**Figure 1:**
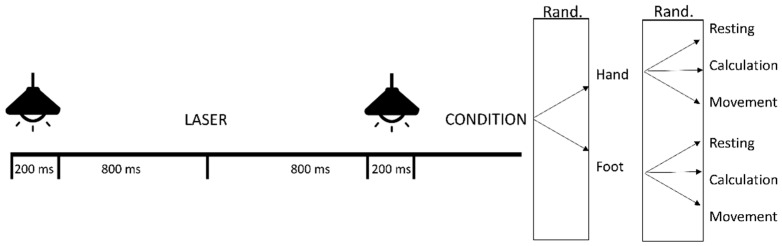
Graphic representation of the experimental setup. A visual stimulus of 200 ms preceded the nociceptive laser stimulus. After 800 ms, a second visual stimulus was provided. The second visual stimulus was followed by a resting period, a movement preparation task or a calculation task (randomised order). Abbreviations: rand: randomisation.

Study participation required one study visit at the Institute of Neuroscience (Université catholique de Louvain, Brussels, Belgium) during which LEPs were recorded. Before the start of the assessment, participants completed a demographic questionnaire, the Pain Catastrophizing Scale (PCS), the Beck Depression Inventory (BDI) and the Pain Disability Index (PDI). Then, the electroencephalographic (EEG) recording started. In total, participants received 180 stimuli, of which 60 were given on the left hand and 60 on the left foot. On each location (hand and foot), three different conditions were applied (resting versus calculation versus movement task). In each condition, three blocks of 10 laser stimuli were delivered. During the rest task, no specific action was required from the participants. During the calculation task, participants performed a mental calculation task out loud (counting backwards from 1000 to 0 in steps of 9). The calculation task was added to the protocol, to evaluate the effect of distraction. During the movement task, participants raised the right index finger as fast as possible. Participants were informed about the type of the required task at the beginning of each condition. A randomisation procedure was used whether the conditions of the hand or foot were tested first. Further within the locations, the order of the three experimental conditions (containing 30 stimuli) was also randomised using a simple randomisation procedure (throwing a dice) [29]. A schematic overview of the experimental setup with the different stimuli and interval times can be found in Figure 1.

A visual warning signal (i.e., a light diode) lasting 200 ms preceded each laser stimulus and was initiated 1 s before stimulus administration (Figure 1). Eight hundred ms after the laser stimulus, a second visual signal of 200 ms was delivered. The first visual stimulus represented a warning stimulus and the second visual stimulus an imperative stimulus [11,12,30]. After this second visual stimulus the action depended on the test task. The inter-stimulus interval ranged between 10 and 15 s in order to avoid habituation effects to nociceptive stimulation. The protocol of the current study is based on the study of Le Pera et al. (2007) among healthy volunteers [12].

At the end of each block, participants were asked to rate the intensity elicited by the laser stimuli using a visual analogue scale (VAS) ranging from “no detection” (VAS = 0) to “maximum pain” (VAS = 100) at the appropriate ends by drawing a vertical line on separate 100-mm horizontal lines. At the middle of the scale (VAS = 50) an anchor marked the borderline between non-painful and painful domains of sensation [31]. Two-factor mixed design ANOVAs were used to compare VAS values between the three conditions and populations.

### 2.3. Sample Size Calculation

An a priori sample size calculation was performed for the entire protocol, based on results from previous studies [12,27]. At least 20 subjects per group (total of 60 subjects) were required for reaching an effect size of 0.5 with a repeated measures ANOVA at the 5% level with a power of 80%.

### 2.4. Demographic Characteristics

Information on participant’s age, sex, time since accident (cWAD), pain level and medication use were evaluated. Medication is divided in non-opioids, opioids, tricyclic antidepressants and selective serotonin reuptake inhibitor (SSRI)/serotonin and norepinephrine reuptake inhibitor (SNRI). Pain intensity at the moment of study participation and pain intensity during the last seven days were measured using a VAS (i.e., by drawing a vertical line on separate 100-mm horizontal lines).

### 2.5. Questionnaires

The Pain Catastrophizing Scale was used to measure participant’s level of pain catastrophizing thoughts. This questionnaire consists of 13 pain-related cognitive items that need to be scored on a 5-point Likert scale (0 = not at all, 4 = all the time) [32]. Scores ≥30/52 indicate a clinically relevant level of pain catastrophizing [33]. The internal consistency, test-retest reliability and validity are found to be acceptable [34,35].

The Beck Depression Inventory was used to evaluate depressive thoughts. Total score ranges between 0 and 63, with a higher score indicating more severe depression. This questionnaire is a reliable and valid tool for the assessment of depressive symptoms in chronic pain patients [36].

The Pain Disability Index provides an indication of the impact of pain on daily living activities. This questionnaire consists of 7 items scored on an 11-point Likert scale (0 = no disability, 10 = completely disabled). Total scores range from 0 to 70, with a higher score indicating higher levels of perceived disability. Differences of 8.5 to 9.5 points are considered to be clinically relevant [37]. The Dutch version of this questionnaire is a valid tool with good internal consistency and test re-test reliability [38].

Total scores on the questionnaires were used in the analyses. Comparisons between patient groups were made with Independent Samples *t*-tests and Mann-Whitney *U* tests depending on the normality and variance of the data.

### 2.6. Laser Stimulation

Laser stimuli were delivered by a CO_2_ laser designed and built in the Department of Physics of the Université catholique de Louvain, Louvain-La-Neuve, Belgium [31]. Stimuli were applied on the dorsum of the left hand (C6-C7 skin dermatomes) and left foot (L5-S1 skin dermatomes). The CO_2_ laser system generates a highly collimated infrared beam (wavelength: 10.6 um). The power output is continuously adjustable between 1 and 25 W. Heat pulse duration was 20 ms. Laser beam diameter was 4 mm. The laser stimulus is highly reproducible (variation <1%). The stimulation site was visualised with a He-Ne laser beam aligned with the CO_2_ laser beam. To avoid skin burns and nociceptor fatigue [39], the location on the skin where the laser stimulus was provided, was moved slightly between 2 successive stimulations. For each subject, the intensity threshold to elicit detections related to the activation of Aδ fibers was determined by measuring the reaction time to the laser stimulation [40]. A series of increasing and decreasing stimulus intensities were delivered to the left-hand dorsum. Participants were asked to push a button as fast as possible when they felt the laser stimulation. Laser stimulus intensity was set at the intensity that repetitively provoked a reaction time below 600 ms (minimum 3 consecutive stimuli). With this procedure, the stimulus intensity was supraliminal for Aδ fiber activation, as confirmed by the reaction times compatible with peripheral nerve conduction velocities within the range of myelinated small fibers. This preliminary session of stimulation was only applied to the dorsum of the left hand since it had been shown with healthy subjects that the threshold for Aδ-nociceptors was somewhat lower at the foot dorsum [41]. Thus, a supramaximal stimulus intensity for activation of Aδ-nociceptors at the level of the hand should be adequate for the activation of Aδ-nociceptors at the level of the foot dorsum.

### 2.7. LEP Recording

LEPs were recorded from 32 Ag-AgCl scalp electrodes, placed according to the International 10–20 system for electrode positioning. Signals were digitized at a sampling rate of 1000 Hz. The signals were referenced to the average of all scalp electrodes. Eyeblinks were simultaneously recorded with a pair of surface electrodes positioned diagonally over the right eye. Electrode impedance was kept below 10 kΩ with a target below 5kΩ. During the assessment, participants were seated in a comfortable chair in a silent room. They were asked to relax muscles, sit as still as possible and gaze at a light diode.

### 2.8. Time-Domain Analysis of LEPs

Offline data pre-processing was performed with the Letswave 6 EEG toolbox (http://letswave.org). EEG recordings were filtered (0.3–30 Hz, Butterworth filter) and segmented into epochs of 6 s (−3 to +3 s relative to the laser stimulus onset). Electroculographic artefacts were removed using Independent Component Analysis. Independent Components having a frontal scalp distribution and a time course compatible with eyeblink artefacts were removed (1–5 ICs). Afterwards, all epochs were visually inspected to remove remaining artefacts (± 100 µV). Finally, epochs were baseline corrected, using the time interval from −1.5 to −1 s as reference (i.e., before the onset of the warning visual stimulus). For each subject, epochs were averaged according to the location (foot versus hand) and condition (rest versus movement versus calculation). LEP components were identified on the basis of their latency and polarity and labelled according to Valeriani et al. 2012 [26]. Peak latencies of N2 and P2 components amplitudes were measured at the vertex (Cz) and defined as the largest negative and positive deflection, between respectively 150–350 ms and 300–500 ms [42]. The LEP N1 component was evaluated at the T8 electrode with the Fz electrode as reference within a targeted time frame of 100–300 ms post-stimulus [42,43].

The amplitude and latencies of the N1, N2 and P2 waves of LEPs obtained following stimulation of the hand and foot during the three conditions (rest, calculation and movement task) between the three groups (HC, CFS, and cWAD) were compared with two-factor mixed design ANOVAs. In addition to comparing latencies and amplitudes of the N1, N2 and P2 waves of LEPs, also the entire LEP waveforms obtained in the different groups were compared using point-by-point ANOVAs. These were performed separately in each patient group for each location to evaluate the effect of the different conditions within that specific group (total number of tests: 6). The steps are described in van den Broeke et al. [44,45,46] and briefly reviewed here. As a first step, LEP waveforms of the three conditions were compared by a point-by-point F-statistic. Then, adjacent samples in time above the critical F-value for parametric two-sided tests were identified and clustered. Additionally, an estimate of the magnitude of each cluster was calculated by summing up the F-values constituting each cluster. Then, a reference distribution of maximum cluster magnitude was obtained by random permutation testing (100 times) of the LEP waveforms of the different conditions. The last step entails calculating the proportion of random partitions that has a larger cluster-level statistic than the observed one. Post-hoc testing with paired-*t*-tests was applied to determine which condition was significantly different in each patient population. This analysis was conducted separately for hand and foot stimulation, as the latencies of the elicited responses may be expected to differ when stimulating the hand and foot, because of the differences in peripheral conduction distance. Clusters were considered significant if *p* <0.05.

### 2.9. Time-Frequency Analysis of LEPs

A time-frequency analysis of the recorded EEG signals was performed to characterize and compare non-phase-locked stimulus-induced changes in the power of ongoing EEG oscillations. A short-time fast Fourier transform (STFFT) [47] with a fixed Hanning window of 500 ms was used. The analysis was performed using the signal recorded at Cz vs. average reference and T8 vs. Fz. The obtained single-trial time-frequency maps were then averaged across trials. A baseline correction was then performed using the interval ranging from −1750 to −1250 ms relative to stimulus onset (i.e., before the onset of the first visual stimulus), to identify decreases (event-related desynchronisation, ERD) and increases (event-related synchronisation, ERS) of oscillation amplitude relative to baseline [48].

Comparison of the time-frequency maps obtained in the different groups was performed using 6 point-by-point ANOVAs (without permutation testing) to evaluate differences in condition within each patient group. In parallel to the analysis in the time-domain, this analysis was conducted separately for hand and foot stimulation. Clusters were considered significant if *p* < 0.05.

All statistical analyses were performed with Letswave 6/7 and R Studio version 0.99.903. Normality was controlled with the Shapiro Wilk test and QQ-plots and equality of variances by Levene’s tests.

## 3. Results

**Figure 2:**
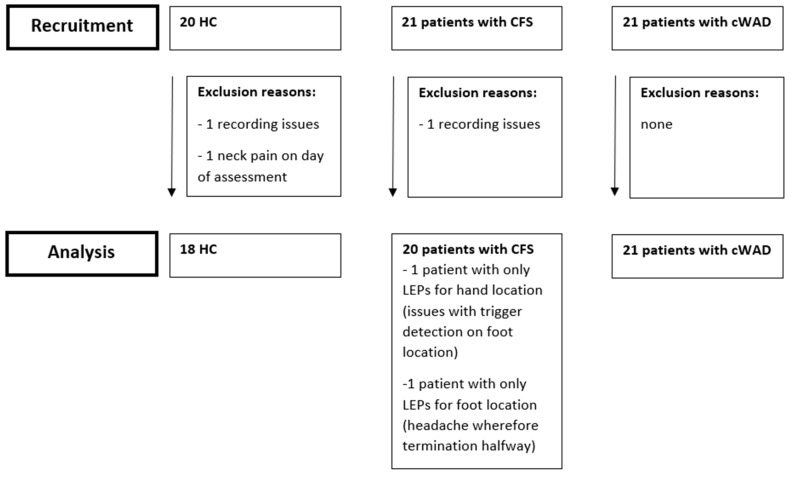
Flow chart of the study. Abbreviations: CFS: chronic fatigue syndrome; cWAD: chronic whiplash associated disorders; HC: healthy controls.

Sixty-two participants (21 patients with CFS, 21 patients with cWAD and 20 HC) took part in this experimental study. Data from one person with CFS and one HC were lost due to recording/processing issues with the EEG data. One HC was excluded after study participation as he reported neck pain (VAS of 31) on the day of testing. One patient with CFS terminated the experiment due to upcoming headache, wherefore not all conditions were present for that patient. For other patients with CFS no triggers were detected during one condition, resulting in data for only one location. In one patient from the CFS group, the EEG signal of one condition contained too many artifacts. As a consequence, we interpolated P7 and P8 with 3 neighbouring electrodes to keep this person in the analysis. Therefore, the analysis was performed on 18 HC, 20 patients with CFS and 21 patients with cWAD. Figure 2 is representing the flow chart of this study.

### 3.1. Group characteristics

Participants had a median age of 46.8 years in the healthy group, 43.4 years in the CFS group and 45.8 years in the cWAD group. In the healthy, CFS and cWAD group, respectively 38.9%, 10% and 52.4% male participants were included. Concerning medication use, in the cWAD group 10 patients took non-opioids (paracetamol, NSAID, benzodiazepine), 3 patients took opioids, 1 patient tricyclic antidepressants and 6 patients SSRI/SNRI. In the CFS group 9 patients took non-opioids (paracetamol, NSAID, benzodiazepine), 1 patient took opioids and 9 patients SSRI/SNRI. Other group characteristics and self-reported measurements are listed in Table 1.

### 3.2. Pain Intensity Ratings

A two-factor mixed ANOVA was conducted to evaluate the effect of condition and population on VAS pain intensity ratings following laser stimulation on both locations. For the hand location, no statistically significant interaction effect was found between population and condition (F = 0.803, *p* = 0.525). Main effect analyses for condition revealed a significant effect (F = 10.366, *p* <0.001), while the main effect for population was not significant (F = 0.450, *p* = 0.640). Simple main effects analysis for condition showed that during the resting condition (41.06 (95% CI 35.85 to 46.27)) VAS ratings were significantly higher than during the counting condition (36.25 (95% CI 30.91 to 41.58)) (*p* <0.001). Pain intensity ratings during the movement condition (40.40 (95% CI 35.26 to 45.55)) were significantly higher than during the counting condition (*p* = 0.001).

For the foot location, no statistically significant interaction between population and condition was found (F = 0.730, *p* = 0.569). Main effect analysis for condition revealed a significant effect (F = 4.675, *p* = 0.012). Main effect for population was not significant (F = 0.353, *p* = 0.704). Simple main effect analysis for condition revealed a higher pain intensity rating for the resting condition (42.22 (95% CI 35.96 to 48.48)), compared to the movement (39.12 (95% CI 32.93 to 45.30)) (*p* = 0.006) and counting conditions (38.66 (95% CI 32.62 to 44.71)) (*p* = 0.028).

### 3.3. Laser-Evoked Brain Potentials: Time Domain Analysis

**Figure 3:**
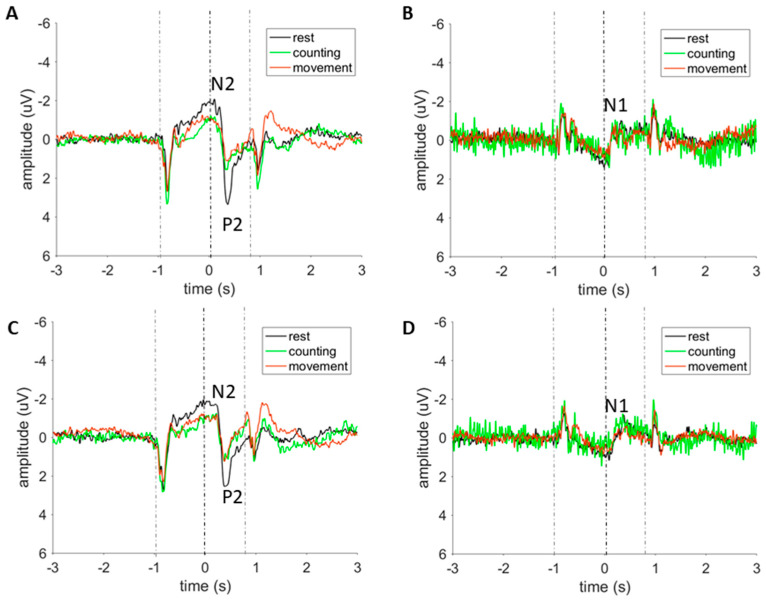
Group-level laser evoked potentials in the time domain, separated by condition. The two gray dotted lines are representing the onset of the visual stimulus. The black dotted line is representing the onset of the laser stimulus. The peaks of the laser-evoked potentials (LEP) are indicated on the figure. (**A**): Average waveforms at Cz (vs. average reference) for hand stimulation. (**B**): Average waveform at T8 (vs. Fz) for hand stimulation. (**C**): Average waveforms at Cz (vs. average reference) for foot stimulation. (**D**): Average waveforms at T8 (vs. Fz) for foot stimulation.

Grand average LEP waveforms recorded at the vertex (Cz) and at the contralateral temporal electrode (T8) are presented in Figure 3. In all three conditions, the laser stimulus elicited a clear negative-positive complex (N2-P2) maximal at the scalp vertex. This complex was flanked by additional responses triggered by the two visual stimuli, which were presented 1000 ms before the laser stimulus and 800 ms after the laser stimulus. The magnitude of the second visual ERP was considerably reduced as compared to the first visual ERP, both for hand and foot location during all conditions. The N2-P2 complex of LEPs was preceded by an earlier N1 wave, less clearly, but visible at electrode T8. Visual stimuli have a dominant negative peak that is highest in the occipital region, while the negative peak for the laser stimulus is highest at the vertex.

**Figure 4:**
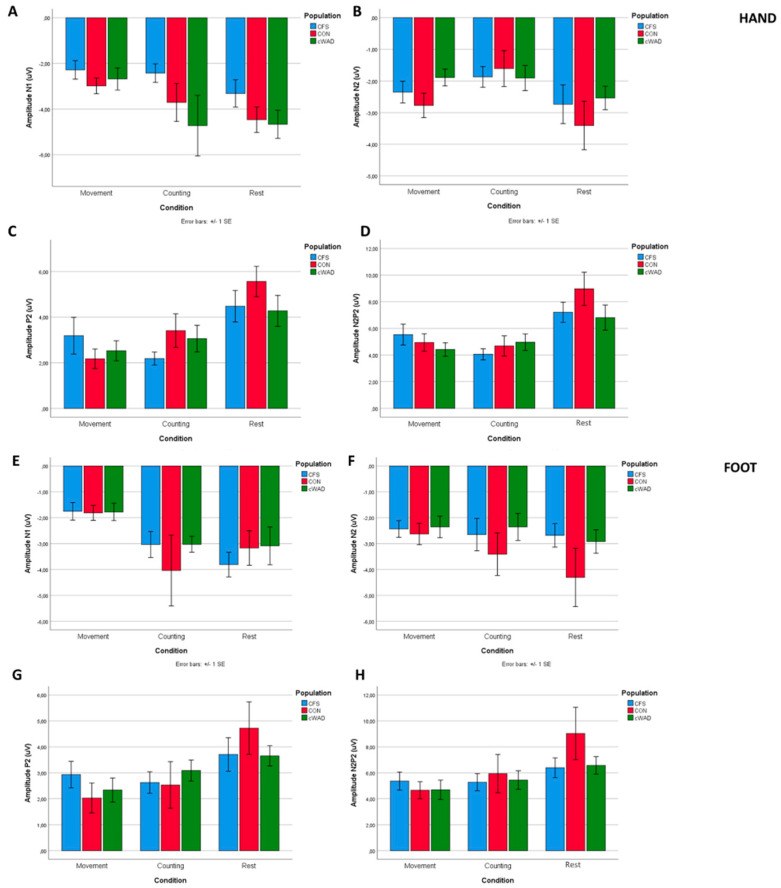
Group-level average magnitude of (LEP) components, separated by type of condition, location and population for nociceptive laser stimulation. Bar plots show the average amplitude of each LEP component. Note that there were no statistically significant differences between populations. (**A**) Magnitude of N1 component for hand stimulation. A significant effect of conditions was found between rest versus movement and between counting versus movement. (**B**) Magnitude of N2 component for hand stimulation. A significant effect of conditions was found between rest versus counting, rest versus movement and between counting versus movement. (**C**) Magnitude of P2 component for hand stimulation. A significant effect of conditions was found between rest versus counting and between rest versus movement. (**D**) Magnitude of N2P2 component for hand stimulation. A significant effect of conditions was found between rest versus counting and between rest versus movement. (**E**) Magnitude of N1 component for foot stimulation. A significant effect of conditions was found between rest versus movement and between counting versus movement. (**F**) Magnitude of N2 component for foot stimulation. (**G**) Magnitude of P2 component for foot stimulation. A significant effect of conditions was found between rest versus counting and between rest versus movement. (**H**) Magnitude of N2P2 component for foot stimulation. A significant effect of conditions was found between rest versus counting and between rest versus movement. Abbreviations: CFS: chronic fatigue syndrome, cWAD: chronic whiplash associated disorders, HC: healthy controls.

The N1 component of the grand-average LEP waveform (averaged across conditions) elicited by stimulation of the hand had an average amplitude of −3.49 µV (SD: 3.1 µV) with a latency of 217 ms (SD: 52 ms) post-stimulus (T8). The N2 was identified at a latency of 216 ms (SD: 95 ms) with an amplitude of −2.3 µV (SD: 2.05 µV) and the P2 was identified at 392 ms (SD: 87 ms) with an average amplitude of 3.4 µV (SD: 2.8 µV) (Cz). The N2-P2 component had an average peak-to-peak amplitude of 5.7 µV (SD: 3.6 µV) (Cz). The N1 component of the LEP waveform measured on the foot had an average amplitude of −2.8 µV (SD: 2.8 µV) with a latency of 223 ms (SD: 51 ms) post-stimulus (T8). The N2 was identified at a latency of 226 ms (SD: 56 ms) with an amplitude of −2.8 µV (SD: 2.6 µV) and the P2 was identified at 403 ms (SD: 61 ms) with an average amplitude of 3.1 µV (SD: 2.7 µV) (Cz). The N2-P2 component had an average peak-to-peak amplitude of 5.9 µV (SD: 4.5 µV) (Cz). Table 2 and Figure 4 present the LEP amplitudes and latencies, separated by condition and stimulus location.

Two-factor mixed ANOVAs were performed to reveal differences in LEP amplitudes and latencies between the three conditions (resting versus counting versus movement preparation) and population (HC versus CFS versus cWAD) (Table 3).

The results of the point-by-point analysis of the LEP waveforms after stimulating the hand in HC revealed two significant clusters at Cz, extending between 294 and 448 ms (*p* = 0.001) and between 468 and 482 ms (*p* = 0.008) post-stimulus. Post-hoc testing with paired-*t*-tests for the first cluster revealed a significant difference between the rest and movement condition (*t* = -4.64, *p* = 0.0002) and between rest and counting (*t* = −2.72, *p* = 0.018). For the second cluster, a significant difference was found between rest and movement (*t* = −4.48, *p* = 0.0004). In the CFS group, a significant cluster was found at Cz between 327 and 449 ms (*p* = 0.0097). Paired *t*-tests revealed a significant difference between rest and movement (*t* = −3.26, *p* = 0.005) and between rest and counting (*t* = −3.39, *p* = 0.004). In the cWAD group, two clusters were revealed, among which one at T8 between 258 and 273 ms post-stimulus (*p* = 0.008) and one at Cz that was extending between 297 and 446 ms post-stimulus (*p* = 0.0126). At T8, a significant difference was revealed between rest and counting (*t* = −2.46, *p* = 0.025) and between counting and movement (*t* = 3.42, *p* = 0.003). At Cz, a significant difference was found between rest and counting (*t* = −3.17, *p* = 0.024) and between rest and movement (*t* = −3.009, *p* = 0.01).

The results of the point −by-point analysis of the LEP waveforms after foot stimulation revealed a significant cluster at Cz between 361 and 523 ms post-stimulus (*p* = 0.014). Post-hoc testing by paired sample-*t*-tests revealed a significant difference between rest and movement (*t* = −2.91, *p* = 0.024) and between rest and counting (*t* = −2.49, *p* = 0.025). In the CFS group, two clusters were found at Cz between 345 and 414 ms (*p* = 0.027) and between 427 and 481 ms post-stimulus (*p* = 0.026). Post-hoc testing indicated one significant difference between rest and counting (*t* = −2.566, *p* = 0.024) which was extending both clusters. In the cWAD group, a significant difference was detected between 345 and 513 ms post-stimulus at Cz (*p* = 0.0007). Post-hoc testing indicated differences between rest and counting (*t* = −2.6001, *p* = 0.044), between rest and movement (*t* = −2.86, *p* = 0.022) and between counting and movement (*t* = −2.52, *p* = 0.025).

### 3.4. Laser-Evoked Brain Potentials: Time-Frequency Analysis

**Figure 5:**
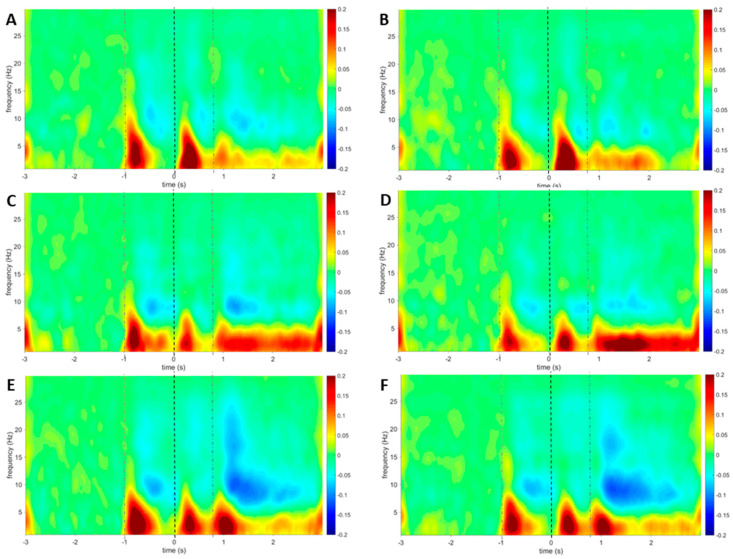
Group level average time-frequency maps in all participants. Visual stimulus onsets (gray dotted lines) and laser onset (black dotted line) are indicated on the figure. The colour bar is representing the amplitude (uV). (**A**): Average time-frequency map at Cz for hand stimulation during resting. (**B**): Average time-frequency map at Cz for foot stimulation during resting. (**C**): Average time-frequency map at Cz for hand stimulation during counting. (**D**): Average time-frequency map at Cz for foot stimulation during counting. (**E**): Average time-frequency map at Cz for hand stimulation during movement preparation. (**F**): Average time-frequency map at Cz for foot stimulation during movement preparation.

The grand average time-frequency maps of the amplitude of ongoing EEG oscillations recorded at the scalp vertex (Cz) are presented in Figure 5. These maps show marked increases of low-frequency activity (<8 Hz) when stimulating the hand between −950 and −550 ms (activity related to the first visual stimulus) and between 150 and 500 ms post-stimulus (activity related to the laser stimulus). Furthermore, a long-lasting increase was revealed from 850 ms post-stimulus onwards. Additionally, a decrease of alpha-band oscillations was observed between −500 and −50 ms and from 1050 ms post-stimulus onwards. For stimulation of the foot, increases of low-frequency activity were revealed between −880 and −650 ms (visual-evoked activity) and between 200 ms and 450 ms (laser-evoked activity). Furthermore, a long-lasting increase was revealed from 950 ms post-stimulus onwards. Additionally, decreases of alpha-band oscillations could be detected between −700 ms and −50 ms and from 1000 ms onwards.

**Figure 6:**
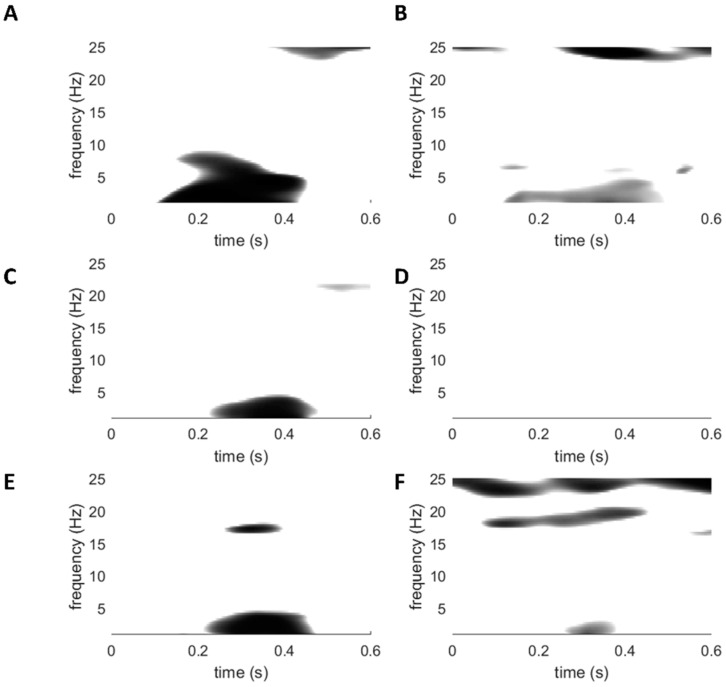
Point-by-point ANOVA testing on group level average time-frequency maps in all participants. (**A**): Anova testing for hand stimulation in HC. (**B**): Anova testing for foot stimulation in HC. (**C**): Anova testing for hand stimulation in CFS. (**D**): Anova testing for foot stimulation in CFS. (**E**): Anova testing for hand stimulation in cWAD. (**F**): Anova testing for foot stimulation in cWAD.

When stimulating the hand, the point-by-point comparison revealed a significant cluster in the HC group at Cz (*p* = 0.001) located at lower frequencies (< 8 Hz) between 200 and 400 ms post-stimulus (Figure 6). Post-hoc testing revealed significant differences between rest and counting (*t* = −3.41, *p* = 0.004) and between rest and movement (*t* = −4.04, *p* = 0.001). Additionally, a significant cluster was revealed around 23–25 Hz from 330 to 550 ms post-stimulus. Post-hoc testing revealed significant differences between rest and counting (*t* = 3.55, *p* = 0.004) and between counting and movement (*t* = −3.52, *p* = 0.003). In the CFS group, a significant cluster was revealed with a *p*-value of 0.008 at Cz between 180 and 380 ms post-stimulus in the lower frequencies (< 5 Hz). Post-hoc testing revealed a significant difference between rest and counting (*t* = −4.82, *p* = 0.001). In the cWAD group, a significant cluster (*p* = 0.003) was found with a frequency below 5 Hz extending between 350 and 450 ms post-stimulus. Post-hoc testing revealed a significant difference between rest and counting (*t* = −3.36, *p* = 0.003) and between rest and movement (*t* = −3.20, *p* = 0.01). A second significant cluster was visible between 225 and 400 ms post-stimulus with a frequency around 17 Hz (*p* = 0.0015). Significant differences were revealed between rest and counting (*t* = 3.18, *p* = 0.005) and between counting and movement (*t* = −3.87, *p* = 0.002).

When stimulating the foot, a significant cluster was revealed (*p* = 0.016) between 110 and 400 ms post-stimulus with a frequency below 5 Hz in the HC group at Cz. Post-hoc testing indicated a significant difference between rest and movement (*t* = −2.56, *p* = 0.022). A second cluster was revealed (*p* = 0.015) with a frequency of around 24-26 Hz between −0.050 and 400 ms post-stimulus. There was a significant difference between movement and counting in the second cluster (*t* = −3.22, *p* = 0.007). No significant clusters were revealed for the CFS group. For the cWAD group, three clusters were revealed at Cz. The first cluster was located at a low frequency (< 5 Hz) between 280 and 315 ms post-stimulus (*p* = 0.012), the second was located at a frequency of around 17–21 Hz (*p* = 0.014) between 70 and 400 ms post-stimulus and the third cluster was located at 22 and 25 Hz (*p* = 0.013), extending between 100 and 400 ms post-stimulus. Post-hoc testing found significant differences between rest and counting (*t* = −2.98, *p* = 0.008) and between rest and movement (*t* = −2.54, *p* = 0.02) for the first cluster. For the second cluster, a significant difference was revealed between rest and movement (*t* = −2.94, *p* = 0.009). Post-hoc testing for the third cluster revealed a difference between counting and movement (*t* = −3.31, *p* = 0.004).

## 4. Discussion

This is the first study that evaluated whether nociception–motor interactions (i.e., the effect of motor activation during motor preparation on the cortical responses to nociceptive stimuli) differ in patients with cWAD and CFS, compared to HC, using LEPs. EEG recordings performed just before the execution of a movement show a negative drift, which is associated with motor preparation and expectancy [10,12]. This slow negative cortical potential, elicited between a warning stimulus and an imperative stimulus, is thought to reflect the engagement of cortical motor areas related to movement preparation [49,50]. In this study, the protocol was constructed in such a way that laser stimuli were provided in a time interval that is able to elicit a contingent negative variation [49]. It was found that motor cortex engagement through movement preparation, exerts an inhibitory effect on the nociceptive system, visible as an attenuation of LEP amplitudes in all three populations. No differences were found in the inhibitory effect through movement preparation between the three populations. Additionally, time-frequency maps revealed significant decreases in amplitude during movement preparation compared to rest.

### 4.1. No Reduced Motor-Induced Inhibition of Nociception in Patients Compared to Healthy Controls

Patients with CFS have been suggested to have reduced exercise-induced endogenous hypoalgesia, i.e., the activation of brain-orchestrated descending nociceptive inhibition in response to exercise [51]. The exact underlying mechanisms of exercise-induced endogenous hypoalgesia remains to be explored. However, several possible explanations such as the hormonal system, cardiovascular system and activation of the primary motor cortex are suggested to play a role in this phenomenon [20]. In this study, we focused on the possible reduced inhibitory effect of engagement of the primary motor cortex on nociception as underlying mechanism. This was evaluated by a movement preparation task namely lifting of the index finger and not during the performance of a real physical exercise. During movement preparation of the right hand, the contralateral cortical motor area is engaged [11], which has neural connections with the anterior cingulate cortex (ACC) [52], possibly leading to cingulate cortex inhibition during this phase. Cingulate cortex inhibition could be responsible for the attenuated N2 and P2 components of LEPs, as these are believed to originate, at least in part, from the ACC [53]. Our finding that motor preparation induced a similar reduction of LEPs in patients with cWAD and patients with CFS compared to HC does not support the hypothesis of reduced motor-induced inhibition of nociception in these patients.

### 4.2. Influence of Movement Preparation on LEP Components

LEPs consist of an early N1 component, which is presumably generated in the primary and secondary somatosensory area and the insular cortex [54,55]. Due to its earlier latency, the magnitude of the N1 component could be more directly related to the ascending nociceptive input than the later N2 and P2 components. Movement preparation is clearly able to reduce the P2 component after nociceptive laser stimulation of the hand and foot. This finding is in line with the findings of Le Pera et al. [12] in healthy volunteers, on which the protocol of the current study is based. Our results also indicate that motor preparation reduced the earlier N1 component. However, a study in 10 healthy volunteers could not reveal an effect of movement preparation on the N1 component [12]. Further research is necessary to unravel the influence of motor cortex activation on the N1 component.

### 4.3. Influence of Distraction on LEP Components

In this study, a mental task (backwards counting) was used to evaluate the effect of distraction. In line with results of previous studies [56,57], N2-P2 amplitude diminished during this mental task by drawing attention away from the LEP stimulus. This effect was detected in all three groups, meaning that shifting attention from a nociceptive stimulus in patients with chronic pain, will attenuate cortical responses in a similar way as in healthy volunteers. It may be possible that the attenuation of LEPs induced by movement preparation, is also due to the distraction effect, wherefore one can probably not solely assign the attenuation of LEPs to motor cortex inhibition.

### 4.4. Time-Frequency Analyses

Brief noxious stimuli are known to elicit increased neuronal activity at frequencies below 10 Hz, between 150 ms and 400 ms post-stimulus [54], which is in line with the results in this study. It is suggested that this activity originates from the sensorimotor cortex, insula, secondary somatosensory cortex and mid-/anterior cingulate cortex [58]. In this study significant differences in transient stimulus-evoked modulations were found between rest and movement preparation in healthy controls and in patients with cWAD in the lower frequencies during this time frame. Based on these results, it might be suggested that besides contextual modulations [59], also movement preparation is able to impact stimulus processing in healthy controls and patients with cWAD. These findings were not found in patients with CFS, potentially revealing another impact of motor cortex activation on stimulus processing in this patient group. This phenomenon is worthy of further confirmation as it could have a potential implication on the construction of rehabilitation strategies for patients with CFS.

### 4.5. Motor Cortex Activation And Pain Relief

Finally, VAS ratings for pain intensity were slightly lower during movement preparation compared to rest when stimulating the foot. However, pain intensity during movement preparation still remains categorised as moderate pain according to the ICD-11 [60]. In a similar experiment with motor cortex activation in patients with fibromyalgia, VAS ratings did not differ between a rest condition and movement task [61], which is in line with the unchanged VAS ratings during stimulation of the hand. Nevertheless, repetitive transcranial magnetic stimulation over the motor cortex is associated with a significant pain relief [62]. Previously, a hypothesis was raised that the analgesic effects induced by experimental stimulation of the motor cortex could be allocated to actions far from the stimulation site [63]. Apparently, the motor cortex activation induced by a simple task such as tapping or lifting a finger is not able to reduce the pain-related cortical responses [61]. Presumably, more physically demanding task are necessary to activate the remote processes that are related to motor cortex activation and responsible for the pain-relieving effect.

### 4.6. Clinical Implications

Patients with cWAD often present themselves with motor system dysfunctions in clinical practice [64]. Impaired cervical movement control is also well-documented in these patients [65,66,67]. Additionally, a broad range of studies explored altered central pain processing in patients with cWAD [68,69,70] and CFS [51,71]. However, studies evaluating the interaction between both systems are scarce. Therefore, this study focused on nociception–motor interactions and could not reveal differences compared to healthy volunteers when evaluating the effect of movement preparation after nociceptive laser stimulation. Moreover, nociception–motor interactions can be activated when preparing a movement on the contralateral side of the painful area, but also on a remote side. The finding of similar responses in healthy volunteers and patients with cWAD and CFS further supports the use of regular body movement and exercise interventions for managing these conditions (due to their inhibitory effect on pain), which is in line with current treatment guidelines for both conditions [72,73,74,75].

### 4.7. Study Limitations

Previous studies already revealed that the motor cortex is engaged during the performance of movements as well as during the movement-preparation phase [11]. However, future studies could use a verification tool (for example measuring motor-evoked potentials) to ensure motor cortex engagement after movement. Alternatively, non-invasive brain stimulation techniques such as transcranial direct current stimulation could be used to more directly evaluate motor cortex inhibition of nociception in these populations. Additionally, the latency of the N1 LEP component elicited by stimulation of the foot is shorter than the latency of the N1 component by stimulation of the hand. Previously, it was already stated that the N1 LEP component to foot stimulation is difficult to detect, which might have influenced these results [55]. To counter this aspect, both regular peak analyses as well as point-by-point analyses were performed with results pointing into the same direction. In this study, there was no equal sex distribution, with a male/female ratio in favor of females in the CFS group. This inequality is in line with the knowledge that CFS primarily affects women with percentages ranging from 65% up to 80% in favor of females [76]. A study in healthy volunteers concluded that there are no differences in amplitude nor latency of LEPs according to sex [77]. However, more recently, a smaller study found lower LEP amplitudes in males compared to females [78]. Future studies are needed to elucidate the exact role of sex on LEPs. Furthermore, patients received no specific instructions about medication use (except for a discontinuation of non-opioid analgesic and anti-inflammatory drugs 48 h before testing) and continued to take their current medication due to ethical considerations and to avoid withdrawal symptoms. Potentially this could have influenced our results since previous studies already revealed an opioid-related N1, N2, and P2 amplitude reduction [79,80,81], as well as a decreased P2 amplitude caused by non-opioids [82]. 

## 5. Conclusions

Movement preparation induced an attenuation of the LEP waveform after nociceptive laser stimulation of the hand and foot. No significant differences in nociception–motor interactions, evaluated by activating the motor cortex through voluntary movement preparation, were found between a group of patients with cWAD, CFS, and healthy volunteers.

## Figures and Tables

**Table 1:** Demographics of all participants separated by population.

	HC (*n* = 18)	CFS (*n* = 20)	cWAD (*n* = 21)	Test Statistic	*p*-Value	Post-Hoc
Age (years)	46.8 (27.7–51.2)	43.8 (35.9–48.4)	45.8 (40.4–51.1)	χ^2^(2) = 1.38	^a^ 0.501	
Sex	M: 7 (38.9%) F: 11 (61.1%)	M: 2 (10%) F: 18 (90%)	M: 11 (52.4%) F: 10 (47.6%)	χ^2^(2) = 8.50	^b^ 0.014	CFS vs. cWAD: 0.009
VAS current pain (mm) (0–100)		49 (19–54.5)	28 (20–53)	W = 213	^c^ 0.507	
VAS last 7 days (mm) (0–100)		50 (31.25–61)	51 (35–62)	W = 179	^c^ 0.79	
PCS (0–52)		17.95 (11.73)	18.1 (10.69)	t(34.6) = 0.133	^d^ 0.895	
BDI (0–63)		16 (11–22)	11 (10–20.5)	W = 131	^c^ 0.332	
PDI (0–70)		38.9 (11.46)	35 (14.34)	t(36.9) = −0.721	^d^ 0.475	
Time since accident (years)			4.7 (4.34)			

For age, visual analogue scale (VAS) pain and the BDI median values with Q1 and Q3 are reported. For sex the exact counts with percentages are reported. For the time since accident, PCS and PDI mean values with standard deviations are provided. ^a^ Kruskal Wallis test; ^b^ Chi-square test; ^c^ Mann-Whitney *U* test; ^d^ independent *t* test; Abbreviations: BDI: Beck Depression Inventory; CFS: chronic fatigue syndrome; cWAD: chronic whiplash associated disorders; HC: healthy controls; PCS: pain catastrophizing scale; PDI: pain disability index; VAS: visual analogue scale.

**Table 2:** LEP latencies, amplitudes and VAS ratings, separated by type of condition, location and population for nociceptive laser stimulation.

	Resting Condition						Movement Preparation Condition						Calculation Condition					
	Hand			Foot			Hand			Foot			Hand			Foot		
	HC (*n* = 18)	CFS (*n* = 19)	cWAD (*n* = 21)	HC (*n* = 18)	CFS (*n* = 19)	cWAD (*n* = 21)	HC (*n* = 18)	CFS (*n* = 19)	cWAD (*n* = 21)	HC (*n* = 18)	CFS (*n* = 19)	cWAD (*n* = 21)	HC (*n* = 18)	CFS (*n* = 19)	cWAD (*n* = 21)	HC (*n* = 18)	CFS (*n* = 19)	cWAD (*n* = 21)
**N1 amplitude (µV)**	−4.47 (2.37)	−3.32 (2.61)	−4.67 (2.81)	−3.17 (2.83)	−3.81 (2.07)	−3.09 (3.35)	−2.98 (1.46)	−2.28 (1.77)	−2.68 (2.24)	−1.81 (1.23)	−1.75 (1.48)	−1.78 (1.55)	−3.71 (3.53)	−2.42 (1.77)	−4.73 (6.09)	−4.04 (5.81)	−3.03 (2.19)	−3.03 (1.41)
**N2 amplitude (µV)**	−3.41 (3.25)	−2.73 (2.67)	−2.54 (1.70)	−4.31 (4.77)	−2.68 (1.98)	−2.92 (2.08)	−2.77 (1.64)	−2.35 (1.50)	−1.89 (1.22)	−2.63 (1.75)	−2.44 (1.40)	−2.35 (1.91)	−1.60 (2.40)	−1.87 (1.42)	−1.91 (1.81)	−3.41 (3.50)	−2.65 (2.73)	−2.35 (2.40)
**P2 amplitude (µV)**	5.56 (2.83)	4.48 (3.02)	4.27 (3.11)	4.72 (4.29)	3.71 (2.82)	3.65 (1.80)	2.17 (1.82)	3.18 (3.50)	2.52 (2.01)	2.03 (2.44)	2.93 (2.23)	2.34 (2.12)	3.41 (3.12)	2.18 (1.23)	3.06 (2.68)	2.53 (3.81)	2.62 (1.81)	3.08 (1.86)
**N2P2 amplitude (µV)**	8.97 (5.29)	7.21 (3.29)	6.81 (4.32)	9.03 (8.57)	6.39 (3.31)	6.57 (3.09)	4.94 (2.78)	5.54 (3.41)	4.41 (2.31)	4.66 (2.79)	5.37 (3.05)	4.69 (3.40)	4.68 (3.21)	4.05 (1.82)	4.96 (2.84)	5.94 (6.25)	5.28 (2.87)	5.44 (3.27)
**N1 latency (ms)**	252 (56)	216 (54)	225 (65)	235 (46)	214 (45)	227 (40)	214 (52)	204 (46)	208 (54)	226 (51)	218 (59)	210 (54)	200 (45)	209 (45)	222 (42)	231 (47)	220 (61)	225 (62)
**N2 latency (ms)**	229 (54)	241 (46)	225 (51)	227 (59)	252 (45)	232 (64)	217 (57)	243 (54)	175 (24)	227 (52)	222 (51)	212 (50)	191 (46)	207 (53)	214 (41)	228 (50)	231 (65)	208 (60)
**P2 latency (ms)**	374 (59)	388 (55)	388 (68)	404 (53)	414 (53)	414 (51)	359 (44)	415 (73)	435 (18)	406 (62)	412 (63)	383 (63)	371 (68)	395 (81)	396 (66)	402 (58)	389 (90)	405 (56)
**VAS**	38 (19.3)	42 (21.7)	43 (17.6)	39 (22.9)	43 (21.7)	44 (25.5)	37 (21.1)	43 (20.1)	41 (16.8)	34 (24.1)	41 (20.5)	40 (23.3)	32 (22.8)	37 (20.7)	39 (16.8)	35 (24.7)	43 (20.6)	40 (24.1)

Abbreviations: CFS: chronic fatigue syndrome, cWAD: chronic whiplash associated disorders, HC: healthy controls.

**Table 3:** Two-factor mixed design ANOVAs for LEP latencies and amplitudes, separated by type of condition, location and population for nociceptive laser stimulation.

Peak	Interaction Effect (Condition x Population)	Effect of Condition	Effect of Population	Condition Post-Hoc Testing (mean (95% CI)	Post-Hoc Rest vs. Counting (Mean Difference (95% CI))	Post-Hoc Rest vs. Movement (Mean Difference (95% CI))	Post-Hoc Counting vs. Movement (Mean Difference (95% CI))
**HAND**							
**N1 amp**	F = 0.568, *p* = 0.635	F = 3.925, *p* = 0.035 *	F = 2.545, *p* = 0.088	Resting: −4.1 (−4.84; −3.46) Counting: −3.6 (−4.75; −2.49) Movement: −2.6 ( −3.14; −2.16)	−0.53 ( −1.91 to 0.84) *p* = 0.442	−1.50 ( −2.39 to −0.62) *p* = 0.001 *	−0.97 (−1.92 to −0.02) *p* = 0.044 *
**N1 lat**	F = 1.418, *p* = 0.235	F = 3.581, *p* = 0.033 *	F = 0.761, *p* = 0.472	Resting: 231 (215;246) Counting: 210 (199;222) Movement:209 (195;222)	21 (0 to 41) *p* = 0.049 *	22 (4 to 41) *p* = 0.017 *	2 (−15 to 19) *p* = 0.839
**N2 amp**	F = 0.862, *p* = 0.470	F = 6.211, *p* = 0.005 *	F = 0.501, *p* = 0.609	Resting: −2.9 ( −3.57; −2.21) Counting: −1.8 ( −2.30; −1.29) Movement: −2.3 ( −2.72; −1.96)	−1.10 ( −1.86 to −0.33) *p* = 0.006 *	−0.55 ( −1.11 to −0.00) *p* = 0.049 *	0.54 (0.01 to 1.08) *p* = 0.045 *
**N2 lat**	F = 1.017, *p* = 0.381	F = 1.344, *p* = 0.259	F = 1.025, *p* = 0.365				
**P2 amp**	F = 2.668, *p* = 0.037 *	F = 23.574, *p* < 0.001 *	F = 0.232, *p* = 0.794	Resting: 4.8 (3.98;5.56) Counting: 2.9 (2.23;3.54) Movement: 2.6 (1.95;3.30)	1.89 (1.18 to 2.6) *p* < 0.001 *	2.15 (1.51 to 2.79) *p* < 0.001 *	0.26 (−0.44 to 0.96) *p* = 0.461
**P2 lat**	F = 0.812, *p* = 0.485	F = 0.935, *p* = 0.368	F = 2.499, *p* = 0.091				
**N2P2 amp**	F = 2.080, *p* = 0.091	F = 26.724, *p* < 0.001 *	F = 0.447, *p* = 0.642	Resting: 7.7 (6.52;8.81) Counting: 4.6 (3.86;5.27) Movement: 5.0 (4.21;5.72)	3.10 (2.09 to 4.10) *p* < 0.001 *	2.70 (1.78 to 3.62) *p* < 0.001 *	−0.40 (−1.24 to 0.44) *p* = 0.348
**FOOT**							
**N1 amp**	F = 0.597, *p* = 0.616	F = 7.964, *p* = 0.002 *	F = 0.201, *p* = 0.819	Resting: −3.6 ( −4.10; −2.61) Counting: −3.4 ( −4.31; −2.43) Movement −1.8 ( −2.16; −1.40)	0.01 ( −1.13 to 1.15) *p* = 0.986	−1.58 ( −2.25 to −0.91) *p* < 0.001 *	−1.59 (−2.47 to −0.71) *p* = 0.001 *
**N1 lat**	F = 0.226, *p* = 0.914	F = 0.415, *p* = 0.647	F = 0.853, *p* = 0.432				
**N2 amp**	F = 0.805, *p* = 0.523	F = 2.848, *p* = 0.063	F = 1.037, *p* = 0.361				
**N2 lat**	F = 0.663, *p* = 0.609	F = 2.144, *p* = 0.126	F = 0.902, *p* = 0.412				
**P2 amp**	F = 1.473, *p* = 0.217	F = 9.835, *p* < 0.001 *	F = 0.007, *p* = 0.993	Resting: 4.0 (3.22;4.84) Counting: 2.7 (2.06;3.44) Movement: 2.4 (1.83;3.03)	1.28 (0.45 to 2.11) *p* = 0.003 *	1.60 (0.87 to 2.33) *p* < 0.001 *	0.32 (−1.04 to 0.41) *p* = 0.386
**P2 lat**	F = 1.086, *p* = 0.366	F = 0.804, *p* = 0.444	F = 0.06, *p* = 0.942				
**N2P2 amp**	F = 1.931, *p* = 0.118	F = 11.905, *p* < 0.001 *	F = 0.375, *p* = 0.689	Resting: 7.3 (5.89;8.77) Counting: 5.6 (4.41;6.69) Movement: 4.9 (4.08;5.72)	1.78 (0.79 to 2.77) *p* = 0.001 *	2.43 (1.24 to 3.61) *p* < 0.001 *	0.65 (−0.25 to 1.55) *p* = 0.154

Abbreviations. amp: amplitude, lat: latency. * Significant result.
